# Staying Physically Active During the Quarantine and Self-Isolation Period for Controlling and Mitigating the COVID-19 Pandemic: A Systematic Overview of the Literature

**DOI:** 10.3389/fpsyg.2020.01708

**Published:** 2020-08-19

**Authors:** Hamdi Chtourou, Khaled Trabelsi, Cyrine H'mida, Omar Boukhris, Jordan M. Glenn, Michael Brach, Ellen Bentlage, Nick Bott, Roy Jesse Shephard, Achraf Ammar, Nicola Luigi Bragazzi

**Affiliations:** ^1^Activité Physique, Sport et Santé, UR18JS01, Observatoire National du Sport, Tunis, Tunisia; ^2^Institut Supérieur du Sport et de l'Education Physique de Sfax, Université de Sfax, Sfax, Tunisia; ^3^Research Laboratory: Education, Motricité, Sport et Santé, EM2S, LR19JS01, the High Institute of Sport and Physical Education of Sfax, University of Sfax, Sfax, Tunisia; ^4^Department of Health, Human Performance and Recreation, Exercise Science Research Center, University of Arkansas, Fayetteville, AR, United States; ^5^Institute of Sport and Exercise Sciences, University of Münster, Münster, Germany; ^6^Department of Medicine, Clinical Excellence Research Center, Stanford University School of Medicine, Stanford, CA, United States; ^7^Faculty of Kinesiology and Physical Education, University of Toronto, Toronto, ON, Canada; ^8^Institute of Sport Sciences, Otto-von-Guericke University, Magdeburg, Germany; ^9^Department of Health Sciences (DISSAL), Postgraduate School of Public Health, University of Genoa, Genoa, Italy; ^10^Laboratory for Industrial and Applied Mathematics (LIAM), Department of Mathematics and Statistics, York University, Toronto, ON, Canada

**Keywords:** COVID-19, quarantine, psychology, sleep, physical activity

## Abstract

The COVID-19 pandemic has created an unprecedented worldwide public health concern. Characterized by rapid and high frequency human-to-human transmission, the World Health Organization has recommended implementation of public health measures, including isolation of all suspected infectious individuals for a 14-day quarantine period, while governments have introduced “social distancing” and “lock-downs” of varying severity to curtail COVID-19 spread. Recent COVID-19 research further suggests there are major sleep problems and psychological disorders (e.g., stress, anxiety, depression) associated with the reduction of movement and activities, as well as the reduced social interaction. There have been no studies examining the effect of physical activity at home during such periods of isolation. However, based on previous research, potential tactics to overcome these negative effects include home-based exercise, exergaming, dancing to music, and participation in yoga. Adults should accumulate at least 150 min of moderate-intensity and at least 75 min of vigorous-intensity of activity divided in to 5–7 sessions per week. This training volume could be reduced by 30% for children and adolescents if replaced by recess or active play in and around the home. Additionally, exercises should be adapted to the fitness level of the participant and a progressive model of intensity and training volume should be utilized, preferably monitored by telephone applications and wearable sensors.

## Introduction

SARS-CoV-2 is a novel coronavirus which takes the form of an enveloped, positive-sense, single-stranded RNA virus. SARS-CoV-2 generally causes mild infections but on occasion will develop into a severe and even lethal communicable disorder termed COVID-19 (Durham Region Health Department, 2003). SARS-CoV-2 was first identified in Wuhan within the People's Republic of China in late December 2019 (Jiang et al., 2020). It presented as a cluster of atypical pneumonia cases among those attending a wet market, where wild animals are typically slaughtered and sold in unhygienic conditions (Wu et al., 2020).

To the best of our knowledge, this novel coronavirus has spread globally, and is currently circulating in at least 213 countries (World Health Organization, 2020a). This makes control of its diffusion a public health emergency for governments, health authorities, scientists, and citizens of affected countries. Difficulties in counteracting and mitigating the spread and burden of SARS-CoV-2 led the World Health Organization (WHO) to declare a global pandemic on March 11th 2020; shortly afterwards the WHO asked all member states to implement procedures to curb human-to-human virus transmission.

Due to the increasing number of COVID-19 cases, hospital admissions for the majority of affected patients were no longer possible. In turn, isolation of confirmed diagnosed cases (Jiang et al., 2020) combined with social distancing, self-isolation, and community lock-downs have been implemented, demonstrably one of the most effective public health measures and interventions. In many areas, a legally binding quarantine has enabled the confinement of potentially infected subjects; a period of 14 days has been recommended to mitigate additional spread (Jiang et al., 2020).

Previously, quarantine strategies were utilized to contain the “Severe acute respiratory syndrome” (SARS) outbreak, with a recommended 10-day quarantine period (Reynolds et al., 2008). Blendon et al. (2004) reported that there was approximately a 54% compliance with the quarantine. Thus, it appears that similar measures might also be effective in curbing transmission of COVID-19 potentially incubating the virus and significantly flattening the epidemic curve.

Social distancing and self-isolation are characterized by movement restriction and reductions in the number of human-to-human interactions. Hawryluck et al. (2004) noted that there were emotional and psychological issues associated with these quarantine periods. Similarly, Reynolds et al. (2008) observed that quarantined individuals experienced enhanced levels of worry and nervousness.

While the Hubei province lock-down was highly effective in reducing deaths and containing the virus (Tang et al., 2020a,b,c), mathematical modeling has demonstrated that these aggressive and stringent suppression strategies are likely unfeasible and/or unsustainable in Western societies, as they prefer to utilize other measures because of perceived social and economic costs. Nevertheless, the Western mitigation interventions are less effective strategies and multiple social distancing and self-isolation measures may be required to adequately control COVID-19 (Tang et al., 2020a). Ferguson et al. (2020) demonstrated that the pandemic could spread in a series of cyclic waves, lasting up to 18 months. This reality would have worldwide implications, dramatically impacting every aspect of daily life.

Recent epidemiological surveys (Banerjee et al., 2020; Clerkin et al., 2020; World Health Organization, 2020b) reported that the elderly are the most vulnerable subjects, followed closely by those with underlying comorbidities (e.g., due to smoking habits, adiposity, sedentary lifestyle or an impaired immune function). As such, it is paramount to counteract the negative impacts of physical inactivity induced by these COVID-19 mitigation strategies (e.g., social distancing and self-isolation). If high compliance with the implemented public health measures is critical to curb the spread of disease, individuals must find alternative methods of performing normal physical activity to help them overcome the negative effects of quarantine and social isolation.

A recent study, Ammar et al. (2020), utilized the short version of the International Physical Activity Questionnaire (Lee et al., 2011), reporting that home confinement during COVID-19 decreased the quantity of physical activity for all exercise intensities and increased daily sitting time.

Additionally, the authors reported decreases during the confinement in vigorous (38.7 vs. 26.0 min/week) and moderate (32.1 vs. 21.4 min/week) physical activity, walking (37.2 vs. 24.6 min/week), and combined activity (108 vs. 71.8 min/week) (Ammar et al., 2020). Moreover, daily sitting time increased from 5.31 before the confinement to 8.41 h/day during confinement. To the best of our knowledge, no current study has examined the possible relationship between quarantine/social isolation and the undertaking of deliberate physical activities within the home environment during these conditions. Nevertheless, some practical recommendations can be made. Therefore, the purpose of this systematic review is to summarize the existing literature regarding the effect of quarantine and social distancing on psychology and well-being, while providing possible practical recommendations for being more physically active during isolation periods.

## The Effects of Quarantine and Social Distancing on Human'S Mental Health

To identify articles evaluating the effects of quarantine and social distancing on mental and physical health, a literature search was performed on May 30th 2020 using two databases: PubMed and Web of Science. The following combination of keywords was used: [(stress) OR (depression) OR (anxiety) OR (psychology) OR (psychiatry) OR (mental health)] AND [(social distancing) OR (lock-downs) OR (self isolation) OR (quarantine)] AND [(novel coronavirus) OR (COVID-19) OR (nCoV)]. In addition, medical subject headings (MeSH) terms were used where appropriate.

Only original articles written in English and accepted for publication in peer-reviewed journals were considered. No restrictions were applied regarding study design, setting, country, or time frame.

Excluded articles were descriptive or review articles, commentaries, correspondence, conference proceedings, letters to the editor, editorials, opinions, and articles (i) with no full text available (ii) based on either health care workers, pregnant women or subjects with a chronic psychiatric illness, cognitive impairment, or infected by COVID-19, and (iii) evaluating a subject's mental health when quarantine was not mandatory. Also articles not written in English were not retained in the present systematic review.

**Figure 1:**
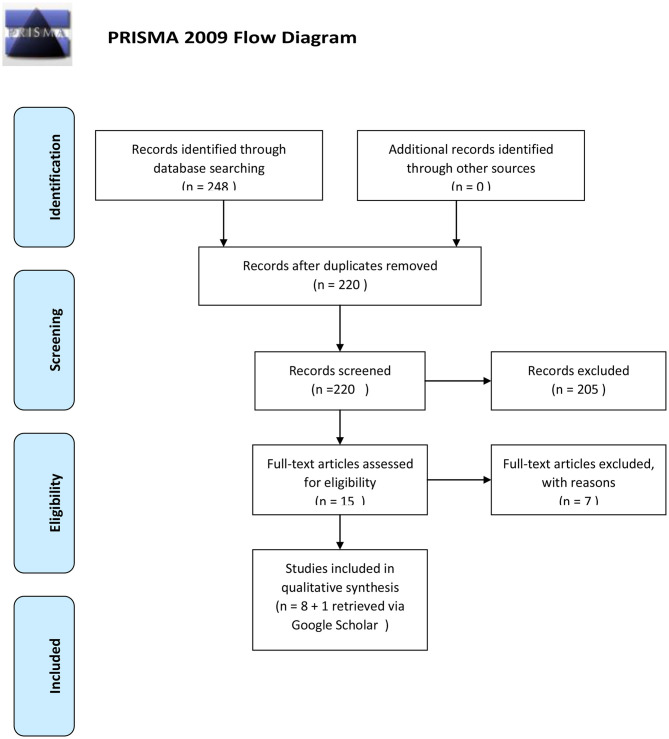
The retrieval and selection process of studies adopted in the present systematic review and meta-analysis.

The initial search revealed 248 results of which 220 remained after removing duplicates. After screening titles and abstracts, 205 articles were excluded. After a careful review of the 15 full text articles, eight were included. An additional search using Google Scholar added one final article, for a total of nine included reports (Figure 1).

The characteristics of each of the nine studies are presented in Table 1. The COVID-19 outbreak impaired individuals' mental health and the quarantine period resulted in anxiety, stress, and depression in individuals independent of geographical location. Several factors are proposed to explain these mental health problems, such as ambiguity and limited information about COVID-19 (Ahmed et al., 2020), media influences (Mazza et al., 2020; Roy et al., 2020), and increased perception of harm to physical, social, and financial health (Lei et al., 2020; Tull et al., 2020). It is noteworthy as the prevalence of mental health symptoms could increase as confinement continues (Ozamiz-Etxebarria et al., 2020). Moreover, the prevalence of psychological disorders is dependent on the number of infected individuals. For example, Ahmed et al. (2020) revealed that psychological disorders were more pronounced in the COVID-19 epicenter (central China's Hubei Province), than other regions of China.

**Table 1:** The COVID-19 outbreak and individuals' mental health.

**References**	**Country**	**Population**	**Gender**	**Methodology**	**Study instruments**	**Results**
Ahmed et al. (2020)	China	1,074 including 674 from Hubei and 396 from other regions	46.8% females and 53.2% males	-Cross sectional -Online survey	The Beck Anxiety Inventory (BAI), the Beck Depression Inventory-II (BDI-II), the Alcohol Use Disorder Identification Test (AUDIT), and the Warwick Edinburgh Mental Well-being Scale (WEMWBS)	-Higher rate of anxiety, depression, hazardous, and harmful alcohol use, and lower mental well-being than usual ratio -Young peoples (21–40 years) are in more vulnerable position in terms of their mental health conditions and alcohol use -Psychological disorders are more pronounced in Hubei province
González-Sanguino et al. (2020)	Spain	3,480 participants	75% females and 25% males	-Cross sectional -Online survey	The Patient Health Questionnaire-2 (PHQ-2), the Generalized Anxiety Disorder Scale-2 (GAD-2), the UCLA Loneliness Scale (UCLA-3), the Multidimensional Scale of Perceived Social Support (EMAS), the Civilian version of the Post-traumatic Stress Disorder Checklist-Reduced version (PCL-C-2), the Functional Assessment of Chronic Illness Therapy Spiritual Well-Being (FACIT-Sp12), the Day-to-Day Discrimination Index (InDI-D), the Self-Compassion Scale (SCS), and the sense of belonging	-18.7% of the sample disclosed a possible diagnosis of depression and 21.6% was likely to be potentially diagnosed with anxiety -Women, younger people, people with previous diagnoses and those who showed symptoms or had a close relative with the disease showed a greater psychological impact -The greatest protector for symptomatology was spiritual well-being -Loneliness was the strongest predictor of depression and anxiety
Lei et al. (2020)	China	420 affected by quarantine and 1,173 unaffected	61.3% females and 38.7% males	-Cross sectional -Online survey	The self-rating anxiety scale (SAS) and the self-rating depression scale (SDS)	-The prevalence of anxiety and depression was ~8.3 and 14.6%, respectively, and the prevalence in the affected group was significantly higher than that in the unaffected group (12.9 vs. 6.7% for anxiety and 22.4 vs. 11.9% for depression) -Lower average household income, lower education level, having a higher self-evaluated level of knowledge, being more worried about being infected, having no psychological support, greater property damage, and lower self-perceived health condition were significantly associated to higher scores of SAS and SDS
Mazza et al. (2020)	Italy	2,766 participants	71.7% females and 28.3% males	Cross sectional -Online survey	The Depression, Anxiety and Stress Scale (DASS-21) and the Personality Inventory for DSM-5–Brief Form–Adult (PID-5-BF)	-In relation to depression, 67.3% of respondents had an average level, 17% were in the high range, and 15.4% were in the extremely high range -In relation to anxiety, 81.3% of respondents had an average level, 7.2% were in the high range, and 11.5% were in the extremely high range-In relation to stress, 72.8% of respondents were in the average range, 14.6% were in the high range, and 12.6% were in the extremely high range -Female gender, negative affect, and detachment were associated with higher levels of psychological distress -Having an acquaintance infected with COVID-19 increased both depression and stress, whereas a history of stressful situations and medical problems raised depression and anxiety levels -Having a family member infected with COVID-19 and being young in age and needing to leave one's domicile to go to work were found to increase anxiety and stress levels, respectively
Ozamiz-Etxebarria et al. (2020)	Spain	976 participants	81.1% females and 18.9% males	-Cross sectional -Online survey: before and after 14th of March	DASS-21	-Levels of symptoms were generally low at the start of the alert -Younger people with chronic diseases reported more symptoms than the rest of the population -Higher levels of symptoms after the stay-at-home order was issued
Satici et al. (2020)	Turkey	1,772 participants	70% females and 30% males	-Cross sectional/ correlational -Online survey	WEMWBS, the Fear of COVID-19 Scale (FCV-19S), the Short Version of the Intolerance of Uncertainty Scale (IUS12), and the Ruminative Response Scale (RRS)	-Intolerance of uncertainty had a significant direct effect on mental well-being -Rumination and fear of COVID-19, in combination, serially mediated the association between intolerance of uncertainty and mental well-being
Tull et al. (2020)	United States	500 participants	47% females and 51.8% males: −0.2% transgender −0.6% non-binary −0.4% other	-Cross sectional -Online survey	DASS-21, the Short Health Anxiety Inventory (SHAI), the Family Economic Strain Scale (FESS), the UCLA Loneliness Scale—Version 3 (ULS-3), and the Multidimensional Scale of Perceived Social Support (MSPSS)	-Being under a stay-at-home order was associated with greater health anxiety, financial worry, and loneliness -The perceived impact of COVID-19 on daily life was positively associated with health anxiety, financial worry, and social support, but negatively associated with loneliness
Wang et al. (2020)	China	1,210 participants	67.3% females and 32.7% males	-Cross sectional -Online survey	DASS-21 and the Impact of Event Scale-Revised (IES-R)	-53.8% of respondents rated the psychological impact of outbreak as moderate or severe -16.5% of respondents reported moderate to severe depressive symptoms -28.8% of respondents reported moderate to severe anxiety symptoms -8.1% reported moderate to severe stress levels
Xiao et al. (2020)	China	170 participants	40.5% females and 59.4% males	-Cross sectional -Online survey	SAS, the Stanford Acute Stress Reaction Questionnaire (SASR), the Pittsburgh Sleep Quality Index (PSQI), and the Personal Social Capital Scale (PSCI-16)	-Anxiety was correlated positively with stress and negatively with sleep quality and social capital -Social capital positively correlated with sleep quality

Implementation of effective measures (e.g., a reduction of population mobility, strengthening individual protection, public education, reduced gatherings) could decrease the prevalence of mental health problems (Qiu et al., 2020). Wang et al. (2020) reported female gender, having COVID-19 symptoms, being a student, and a poor self-perceived health were associated with higher anxiety and depression. Furthermore, the use of specific measures (e.g., hand-washing) and the availability of accurate information may mitigate these effects (Wang et al., 2020). Mazza et al. (2020) reported that having COVID-19 infected family members or a history of stressful situations and medical problems may increase depression and anxiety levels. Other factors such as lower average household income, lower education levels, higher self-evaluated level of knowledge, being more worried about infection, absence of psychological support, and greater property damage could also increase the prevalence of mental health problems (Lei et al., 2020).

Specific attention should be paid to younger people during the periods of quarantine. For example, Ahmed et al. (2020), González-Sanguino et al. (2020), and Ozamiz-Etxebarria et al. (2020) demonstrated younger people were more vulnerable in terms of their mental health during COVID-19 because of their enhanced access to social media information. Moreover, the new online education environment could exacerbate stress levels in students (Ozamiz-Etxebarria et al., 2020).

Sleep quality may also be negatively impacted during the COVID-19 outbreak. Xiao et al. (2020) examined the link between sleep quality and psychological measures, showing anxiety was associated with impaired sleep.

The previous studies suffer from multiple drawbacks. The snowball method of sample selection is not representative of a country-level population. This strategy does not result in a random sample of the general population. For example, participation of men was lower than that of women in six studies (González-Sanguino et al., 2020; Lei et al., 2020; Mazza et al., 2020; Ozamiz-Etxebarria et al., 2020; Satici et al., 2020; Wang et al., 2020). Furthermore, elderly people were underrepresented in four studies (González-Sanguino et al., 2020; Lei et al., 2020; Ozamiz-Etxebarria et al., 2020; Wang et al., 2020); online surveys have limited the participation of elderly people, who are less likely to utilize technology (e.g., social media). Future studies with greater methodological rigor, particularly around sample selection, are warranted.

Additionally, some published data (González-Sanguino et al., 2020; Tull et al., 2020) refer only to the alarm situation of the first period of quarantine; prospective data, using the same population, is needed to examine changes as the pandemic continues. Despite the inherent limitations in previous studies, it appears that COVID-19 could affect negatively an individual's mental health.

Thus, it is important to identify possible strategies to effectively cope with these psychological issues while simultaneously maintaining the social isolation necessary to reduce the spread of COVID-19. Based on an overview of the existing literature, we suggest home-based exercises and other methods of increasing habitual physical activity, as used for many cardiovascular and mental pathologies, might help in reducing the presently observed negative psychological problems.

## Possible Positive Effects of Being Physically Active During Quarantine and/or Social Isolation

According to Garber et al. (2011) and based on the recommendation of the American College of Sports Medicine, exercise improves physical and mental health and/or fitness in most persons irrespective of their training habits. They indicate that flexibility, cardiorespiratory, resistance, and neuromotor exercises are recommended for all ages of healthy adults. It has been reported that home-based tactics could include aerobic activities, balance and flexibility exercises, and muscular strength and endurance training, among others (Almeida et al., 2019). Such tactics have been widely utilized to improve both cardiovascular and psychological parameters in a variety of pathologies and co-morbidities (Almeida et al., 2019). For example, Loh et al. (2019) reported a positive effect of a home-based exercise program on anxiety, mood, and social and emotional well-being among older patients with cancer. Likewise, Chien et al. (2011) reported a significant positive effect of an individualized home-based physical program on functional exercise capacity and health-related quality of life in people with chronic heart failure. Given the increased risk that individuals with comorbid conditions face in the wake of COVID-19, these home-based physical activity tactics provide a safe alternative to classic societal options such as public gyms or in-person group-based fitness classes.

Another potentially beneficial activity during the quarantine period could be the utilization of exergames (i.e., active video games) (Viana and de Lira, 2020). Such games allow individuals to be physically active through their interactions with action and motion sensors (Viana and de Lira, 2020; Viana et al., 2020). Widely utilized exergames simulate swimming, rowing, cycling, running, and walking, using visual and auditory stimuli with equipment that includes remote controls with accelerometers, cameras, and heart rate monitors (Viana and de Lira, 2020; Viana et al., 2020).

Exergames increase both motivation and self-efficacy, thus encouraging physical activity (Stenström et al., 1997). Exergames can be played with a partner and may provide opportunities for interaction and communication. Consequently, this may improve psychosocial well-being (Xu et al., 2016). However, partner preferences must be considered, particularly for elderly people. Xu et al. (2016) recommended pairing young-old (i.e., aged 74 years and below) with adolescents in exergame play to foster intergenerational communication. Additionally, it was recommended to pair the old-old (i.e., aged 75 years and above) with peers to improve their psychosocial well-being.

In healthy populations, Viana et al. (2017) showed that a single 20-min exergame Zumba fitness session, performed at moderate intensity (i.e., 70% of the predicted maximum heart rate, rating of perceived exertion on Borg scale = 12), reduced anxiety levels in young adult women who, as previously mentioned, are more prone to anxiety, depression, and panic disorders during these types of socially isolating situations. However, the previous study suffers from multiple shortcomings (Viana et al., 2017). First, this study lacked a designated control group (e.g., alternative exercise, quiet rest, no exercise condition). Second, physiological (e.g., increased norepinephrine, beta-endorphins, serotonin, increased parasympathetic activity), and/or psychological mechanisms (e.g., increased self-efficacy, a sense of mastery, distraction) responsible for reducing state anxiety were not examined. Furthermore, only acute responses to exergames were examined; consequently, the chronic exergames effects were not identifiable. Based on the findings of the previous study, Viana and de Lira (2020) recommended moderate intensity exergames for reducing anxiety levels.

Interestingly, patients and elderly persons may also benefit from playing exergames. For example, in the functional rehabilitation of Parkinson's disease, Calcagni and Gana (2019) found that exergames had positive effects on perceived stress. Among elderly persons, Rosenberg et al. (2010) showed that exergaming had positive effects on cognitive performance, mental health-related quality of life, and depressive symptoms. Similarly, Byrne and Kim (2019) showed a positive effect of exergames for improving mood disorders.

In a recent systematic review and meta-analysis, Viana et al. (2020) concluded exergame interventions provided within-group improvements in anxiety levels across multiple clinical populations (e.g., subsyndromal depression, multiple sclerosis, fibromyalgia, burns in the upper limbs, heart failure); however, level of improvement did not differ from non-exercise interventions. Furthermore, the results of this meta-analysis should be interpreted with caution due to a limited number of studies, small sample sizes, differing research designs, and differing populations. Additionally, the employed exergame protocols were different, making it impossible to determine the most beneficial recommendations and contributing to the high level of heterogeneity. Moreover, 14 of 15 studies did not report the duration between the last exercise session and when anxiety levels were assessed. As a result, more rigorous scientific work on this topic is warranted.

Individuals can listen to music during exergames as well as during other types of physical activity, such as dancing and Yoga. These latter pursuits, could be undertaken and coached by trainers using video communication. Kalyani et al. (2019) showed that dancing has beneficial effects on psychological symptoms (i.e., anxiety and depression) in people with Parkinson's disease. Dancing can encourage practitioners to express and channel their feelings and emotions, resulting in enjoyment, enhanced motivation, and mood (Prewitt et al., 2017), thus positively impacting their quality of life (Hackney and Bennett, 2014). Furthermore, dance activities require synchronization between action and the accompanying music. As reviewed by Chtourou et al. (2015), music has an ergogenic effect on physical and cognitive performance and might promote motivation and engagement, leading to reduced subjective stress, anxiety, and depression. In a recent systematic review and meta-analysis, Terry et al. (2020) concluded listening to music during physical activity boosted positive affective valence, improved physical engagement, reduced perception of exertion, and enhanced physiological responses. Ghayomzadeh et al. (2019) reported that dance training improved subjective sleep quality and tended to improve some immune parameters (i.e., CD4^+^, T-cell count). Hammami et al. (2020) indicated dance, in the elderly, induced positive functional adaptations (e.g., balance).

Additionally, in a recent systematic review, Wang and Szabo (2020) provided evidence of positive psychophysiological effects of Yoga. Yoga practice improved the sense of well-being by increases in endogenous melatonin secretion (Harinath et al., 2004). The positive effects of Yoga on depression, anxiety, and self-efficacy were supported in the systematic review of Wang and Szabo (2020). It has been reported that 2 months of Yoga reduced perceived anxiety scores in women suffering from anxiety disorders (Javnbakht et al., 2009). Type 2 diabetes patients can also benefit from Yoga, showing a reduced anxiety, insomnia, and depression and an improvement in their overall quality of life (Shiju et al., 2019).

## Who Recommendations to Stay Active in and Around the Home (World Health Organization, 2020c,d)

Physical activity is defined as energy expenditure from body movement including daily life activities (e.g., traveling, transportation, working, playing) (Caspersen et al., 1985; World Health Organization, 2018). However, exercise is defined as planned, structured, and repetitive movements to improve or maintain physical fitness (Caspersen et al., 1985; World Health Organization, 2018). In addition to that, all leisure time physical activity induces supplementary health benefits (World Health Organization, 2018).

To improve or maintain muscular fitness and cardiorespiratory and bone health, the World Health Organization (2018) recommended adults with non-communicable diseases (i.e., over 18 years) perform at least 150 min of moderate-intensity aerobic activities or at least 75 min of vigorous-intensity or combination of moderate- and vigorous-intensity activities. Additionally, WHO recommendations include the inclusion of at least two muscle-strengthening exercises per week for the major muscle groups. Older adults with low levels of mobility should perform at least three physical activity sessions per week. For children and adolescents daily 60 min moderate to vigorous physical activity with at least three muscle-strengthening exercises per week is recommended. For children under 5 years (and over 1 year), daily 180 min moderate to vigorous physical activity is recommended.

Although activity restriction has been imposed during the COVID-19 pandemic, maintaining an active lifestyle is crucial for individuals with current health problems. As a result, the World Health Organization (2020c,d) published the following recommendations to improve total daily activity levels:

For adults: (i) climb stairs as much as possible, (ii) increase household chores, (iii) participate in online physical activity classes coupled with enjoyable music, (iv) perform muscle strengthening exercises, and (v) perform enjoyable physical activity.

For children and adolescents (aged between 5 and 17 years): (i) participate in active play, (ii) join online activity classes, (iii) perform indoor challenging games, (iv) learn new physical skills and (v) perform muscular strength training.

## Recent Recommendations for Physical Activity During the COVID-19 Situation

While performing outdoor physical activity is important, recommendations for home-based exercises during confinement are crucial. Although there is limited support for the idea that being physically active will help reduce disease severity (Kaux and Francaux, 2020), performing exercises while maintaining or even improving health-related physical fitness is better than remaining sedentary (Jiménez-Pavón et al., 2020).

For older individuals, participating in a multicomponent exercise program (i.e., aerobic, balance, coordination, resistance, and mobility training exercises), with the possible inclusion of a cognitive training program is recommended (Jiménez-Pavón et al., 2020). Recommendations include aerobic exercise, 5 days that could be increased to 7 days per week which can be adjusted in terms of volume and intensity (Jiménez-Pavón et al., 2020). The recommended duration is of 50–300 min and may be increased to 200–400 min according to the individual perception of the exercises loads and fatigue (Jiménez-Pavón et al., 2020). Most training should be performed at moderate intensity (i.e., 40–60% heart rate reserve or 65–75% of maximal heart rate) (Jiménez-Pavón et al., 2020). Mobility training should be included in all training sessions (Jiménez-Pavón et al., 2020). The authors suggest that at least two sessions of resistance and balance and coordination training per week should be included (Jiménez-Pavón et al., 2020).

For older adults, Lakicevic et al. (2020) and Hammami et al. (2020) suggest a program of multicomponent exercise (i.e., aerobic, flexibility, balance, coordination, resistance, and mobility training exercises). The recommended duration is between 150 and 225 min (i.e., 150 min of moderate-intensity aerobic exercise and 75 min per week of vigorous intensity aerobic sessions) equally divided between 3–5 days, with at least two resistance training (i.e., 10–15 repetitions for 1–3 sets in 8–10 exercises) and three balance training sessions per week. Ten minutes of stretching per day may also be included.

Ricci et al. (2020) confirmed the World Health Organization (2020c,d) and the previous study recommendations. Additionally, they recommended (i) active short breaks [i.e., 1–2 min break every 30 min (e.g., increasing the home activities: cleaning and gardening, taking the stairs and dancing)], (ii) walking and standing up (e.g., try not to sit and take every chance to stand up and walk), and (iii) joining online physical activity classes (e.g., online virtual exercise classes suitable for endurance, balance, strength, and flexibility).

The most important part of children's activities involve recess, active play, physical education classes, structured sports activities, and active time in playgrounds and parks (Guan et al., 2020). However, during COVID-19 quarantine, several governments have required physical distancing and school closures, increasing sedentary time. As a result, children are demonstrating more sedentary activity with less consistent sleep patterns (i.e., sleeping later) (Guan et al., 2020). Guan et al. (2020) recommended preschool children (aged 3 and 4 years) accumulate more than 180 min physical activity, engage in <60 min sedentary screen time, and have good nightly sleep quantity (i.e., 10–13 h). For school-age children and adolescents (i.e., 5–17 years), they recommended participation in more than 60 min of moderate to vigorous intensity physical activity, engaging in <120 min sedentary recreational screen time, and having a good nightly sleep quantity (i.e., 9–11 h). Also, for children and youth (i.e., 5–17 years), Hammami et al. (2020) recommended 60 min of daily moderate to vigorous aerobic physical activity with three muscle strengthening sessions. Ricci et al. (2020) advised playing video games with motion sensor controls.

With restricted spaces to perform aerobic exercise, Hammami et al. (2020) suggested training sessions could be performed on treadmills and stationary bikes, as well as utilizing rowing ergometers. In addition, dancing and gymnastics are recommended activities for increasing aerobic exercise. The intensity of these exercises could be monitored via heart rate monitors. The exercise intensity is classified as: (i) low at 65% of maximal heart rate (HRmax) for trained/active and 60% of HRmax for sedentary and elderly/patients, (ii) moderate at 80% of HRmax for trained/active, 75% of HRmax for sedentary and 70% of HRmax for elderly/patients, and (iii) high at 90% of HRmax for trained/active, 85% of HRmax for sedentary and 80% of HRmax for elderly/patients.

It is worth noting that sudden engagement in heavy aerobic and resistance training programs is not prudent as these exercises may reduce immune function (Guan et al., 2020). Therefore, the above-cited recommendations should be adapted according to the practitioners fitness level and progression; volume and intensity should be adapted to reduce negative impact on the immune system and to obtain better physical fitness. Telephone applications and wearable sensors may help to control training progression.

On the other hand, as indicated by Woods et al. (2020), participating in any type of physical activity during a systemic viral infectioin should be avoided as the inflammatory reaction in the muscle cells and coronary artery walls increase the risk of sudden cardiac death.

## Practical Recommendations

In the absence of a specific investigation focused on the value of enhanced physical activity during quarantine and social isolation, inferences from earlier research suggest some practical options that can be utilized during these specialized situations:

1. Home-based exercises, such as aerobic activities, balance and flexibility exercises, and muscular strength and endurance training, have the ability to reduce stress, anxiety, and depression during quarantine and social isolation. Adults should accumulate at least 150 min of moderate-intensity and at least 75 min of vigorous-intensity of endurance and aerobic activities, divided between 5–7 sessions per week with 10–20 min of balance and flexibility exercises during each training session (World Health Organization, 2020c). Strength training should be performed at least two times per week (World Health Organization, 2020c). This training volume may be reduced by 30% for children and adolescents if replaced by recess and active play around the home (World Health Organization, 2020d).
2. Using similar training characteristics to increase adherence to physical activity and replacing some of the aforementioned strategies with alternative options could be recommended:
  - Exergames provide an effective tactic to reduce stress, anxiety, and mood disorders during quarantine and social isolation (Viana and de Lira, 2020). This strategy could replace part or a whole session of aerobic or endurance training when the duration of the exergame session is 20 min or more (Viana and de Lira, 2020).
  - Dancing and yoga offer forms of physical activity that can be performed in small areas, and such activities might be helpful in reducing stress, anxiety, and depression, and even improving the quality of sleep (Chtourou et al., 2015; Ghayomzadeh et al., 2019; Kalyani et al., 2019; Wang and Szabo, 2020). These exercises could be performed for >30 min per session.
3. The combination and changes of physical activity types during the week is important for increasing the participant's adherence and the motivation.
4. Exercises should be adapted to the participant's fitness level and progression in the intensity and volume of the training should be utilized. This strategy may potentially be controlled via telephone applications and sensors.

## Perspectives

Based on these hypotheses, researchers should carry out high-quality studies investigating the impact of inactivity related to self-isolation and quarantine during the COVID-19 pandemic and the best ways to mitigate the burden of disease and the psycho-social issues associated with such stringent public health measures.

## Data Availability Statement

The original contributions presented in the study are included in the article/supplementary material, further inquiries can be directed to the corresponding author/s.

## Author Contributions

HC, KT, and NB conceived the paper. All authors drafted and critically revised the paper.

## Conflict of Interest

The authors declare that the research was conducted in the absence of any commercial or financial relationships that could be construed as a potential conflict of interest.
